# IL-17 producing mast cells promote the expansion of myeloid-derived suppressor cells in a mouse allergy model of colorectal cancer

**DOI:** 10.18632/oncotarget.5435

**Published:** 2015-09-26

**Authors:** Xiaowei Chen, Michael J. Churchill, Karan K. Nagar, Yagnesh H. Tailor, Timothy Chu, Brittany S. Rush, Zhengyu Jiang, Edwin B.C. Wang, Bernhard W. Renz, Hongshan Wang, Ming Chiu Fung, Daniel L. Worthley, Siddhartha Mukherjee, Timothy C. Wang

**Affiliations:** ^1^ Division of Digestive and Liver Disease, Columbia University, New York, NY, USA; ^2^ Department of Medicine and Herbert Irving Comprehensive Cancer Center, Columbia University, New York, NY, USA; ^3^ Division of Biology, School of Life Science, The Chinese University of Hong Kong, Shatin, NT, Hong Kong SAR, China

**Keywords:** histamine deficiency, mast cell, IL-17, myeloid-derived suppressor cell, colorectal cancer

## Abstract

Food allergy can influence the development of colorectal cancer, although the underlying mechanisms are unclear. While mast cells (MC) store and secrete histamine, immature myeloid cells (IMC) are the major site of histidine decarboxylase (HDC) expression, the enzyme responsible for histamine production. From our earlier work, we hypothesized that histamine is central to the association between allergy and colorectal carcinogenesis through its influence on the MC-MDSC axis. Here, we show that in wild type (WT) mice, ovalbumin (OVA) immunization elicits a typical T_H_2 response. In contrast, in HDC−/− mice, the response to OVA allergy is skewed towards infiltration by IL-17 expressing MCs. This response is inhibited by histamine treatment. The HDC−/− allergic IL-17-expressing MCs promote MDSC proliferation and upregulation of Cox-2 and Arg-1. OVA allergy in HDC−/− mice increases the growth of colon tumor cells in both the MC38 tumor cell implantation model and the AOM/DSS carcinogenesis model. Taken together, our results show that histamine represses IL-17-expressing MCs and their subsequent activation of MDSCs, attenuating the risk of colorectal cancer in the setting of food allergy. Targeting the MC-MDSC axis may be useful for cancer prevention and treatment in patients, particularly in those with food allergy.

## INTRODUCTION

Although mast cells (MCs) are known for rapid histamine secretion and degranulation in IgE-mediated immunity, it is now appreciated that MCs also contribute to cancer development (reviewed in [1–3]). In particular, MCs influence the polarity of T_H_1, T_H_2, and T_H_17 cells by regulating the maturation of antigen presenting cells [4, 5] and the release of proinflammatory cytokines [3, 6]. Proinflammatory cytokines produced by MCs may promote carcinogenesis through direct tumor stimulation as well as through their support of immune suppressive cells, such as regulatory T (Treg) cells and myeloid-derived suppressor cells (MDSC) [6–9]. MDSCs arise from CD11b+Gr1+ immature myeloid cells (IMC) found within the tumor microenvironment. MDSCs promote carcinogenesis in part through T-cell suppression, which results in a loss in tumor immunity [10]. Recent studies suggest that MCs regulate the recruitment and immunoregulatory activity of MDSCs [11, 12]. MCs also play critical roles in epithelial cell activation, T helper cell polarization and intraepithelial stromal cell remodeling [13–16]. Given these many diverse functions, MCs could play an important role in promoting tumor development.

The intestine contains a broad range of IL-17-expressing cells, such as innate-like T cells, neutrophils, eosinophils, invariant natural killer T (iNKT) cells, αβ and γδ T cells, and lymphoid tissue inducer-like cells (LTi-like) cells [17–20]. Recently, MCs were also shown to produce IL-17 in human cancer [9, 21–23]. IL-17 is a major proinflammatory cytokine, and is known to stimulate myeloid cells, including tumor-promoting MDSCs [24, 25]. The mechanisms involved in the overproduction of IL-17 in the tumour microenvironment, however, have not been fully investigated.

Tissue histamine promotes angiogenesis, neurotransmission, gastric acid secretion, myeloid cell differentiation, and innate immunity [26, 27]. In addition, histamine has a well established role in modulating inflammatory reactions, including intestinal food allergy [31, 32]. MCs store and secret histamine yet are also regulated by histamine [33, 34]. Interestingly HDC, the enzyme generating endogenous histamine, is primarily expressed by CD11b+Gr1+ IMCs, rather than MCs [28]. IMCs are a group of innate immune cells that are often localized adjacent to MCs in the gut, and help to balance immune responses during persistent unresolved inflammation [10, 35]. In previous work, we found that knockout of HDC, the chief enzyme that generates histamine in CD11b+Gr1+ cells, increases MDSCs and tumorigenicity in the azoxymethane (AOM) and dextran sodium sulfate (DSS) model [28]. A number of studies have implicated allergy, a histamine-mediated disorder, in the modulation of cancer risk (reviewed in [29, 30]). We hypothesized that histamine would help protect patients with food allergy from developing colorectal cancer. We found that HDC gene deletion promoted colorectal carcinogenesis through increased MC production of IL-17, leading to an expansion of CD11b+Gr1+ MDSCs and suppression of CD8 cells. Interestingly, this increased tumor susceptibility was rescued by treatment with or replacement of histamine. This study identifies a histamine-dependent link between food allergy and colorectal carcinogenesis, and highlights a new pathway to target in the prevention and treatment of bowel cancer.

## RESULTS

### IL4/IL4Rα axis mediates WT but not HDC−/− OVA intestinal allergy

**Figure 1:**
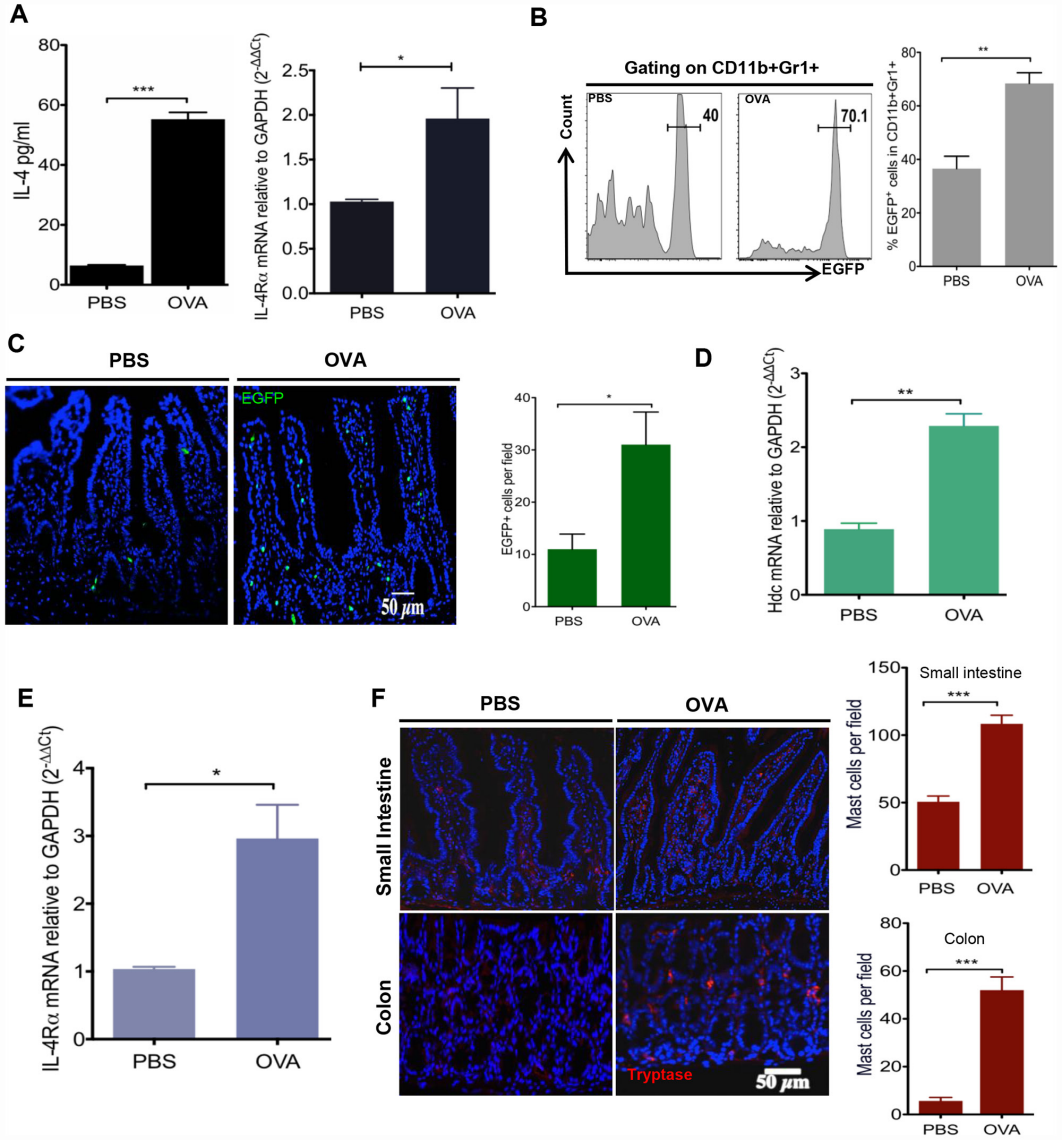
OVA intestinal allergy in WT mice induces the production of IL-4 and accumulation of HDC-expressed CD11b+Gr1+ cells WT mice (*n* = 5/group) were immunized with OVA (30 ug i.g. and 50 ug i.p.) or PBS. **A.** Serum IL-4 levels were quantified by ELISA (Left); Intestinal IL-4Ra expression was exanimated by qRT-PCR (Right). **B.** Representative flow plots of circulating HDC-EGFP+ in blood CD11b+Gr1+ cells (Left). Bar graph (Right) illustrates the proportions of cell populations with statistics. **C.** Imagines show EGFP+ cells from OVA treated and PBS controls small intestine frozen sections (Left). EGFP+ cells were enumerated in each observed microscopic field (Right). Expression of Hdc mRNA **D.** and IL-4Rα **E.** from sorted small intestine CD11b+Gr1+ cells. **F.** Representative images of small intestine and colon immunofluorescence staining for tryptase+ MCs in HDC−/− mice treated with either OVA or PBS (Left). Tryptase+ MCs were counted per microscopic field (Right). Data are representative of at least two independent experiments.

To better understand the interrelationship between histamine-secreting MC and HDC-expressing IMCs in food allergy and colorectal cancer, we treated WT C57BL/6 and HDC-EGFP mice with 30 ug i.g. and 50 ug i.p. of OVA daily for 10 consecutive days [36]. As expected, serum IL-4 levels and small intestinal mRNA expression of IL-4 receptor α chain (IL-4Rα) were significantly increased in OVA allergic mice (Figure 1A). In addition, in OVA treated HDC-EGFP mice, in which EGFP fluorescence identifies CD11b+Gr1+ HDC-expressing cells [28], we detected numerous circulating and intestinal EGFP+ myeloid cells (Figure 1B and 1C). Moreover, HDC mRNA levels were upregulated in sorted small intestinal CD11b+Gr1+ IMCs (Figure 1D).

It has been shown previously that T_H_2 cell derived IL-4 activates CD11b+Gr1+ cells through its receptor, IL4Rα [37, 38]. Consistently, we also detected elevated IL4Ra mRNA levels in small intestinal CD11b+Gr1+ cells of OVA-treated mice compared with PBS treated controls (Figure 1E). A causal link has been suggested between allergic histamine release and cytokine production of T helper cells, given that histamine receptor(s) knockout mice exhibit similar defects in T helper cell activation on common allergic signals [39–41]. To investigate whether histamine deficiency alters myeloid cell activation in the OVA intestinal allergy model, we crossed HDC knockout mice (HDC−/−) with HDC-EGFP and subjected them to the same OVA regimen. Although HDC−/− mice showed an attenuated OVA IgE response (Supplementary Figure S1A), surprisingly HDC-EGFP+ IMCs in the small intestine remained highly abundant (Supplementary Figure S1B), despite the absence of any increase in serum IL-4 levels (Supplementary Figure S1C). The CD11b+Gr1+ cells in the HDC−/− mice functionally resembled MDSCs, with 10-fold greater expression of Arginase 1 (Arg-1) compared to CD11b+Gr1+ cells from WT mice treated with the same OVA regimen (Supplementary Figure S1D). The differences between WT and HDC−/− mice in response to OVA immunization suggested that histamine might mediate the activity of HDC−/− CD11b+Gr1+ cells. This led us to investigate MCs, the principal histamine secreting cell type. In the non-immunized (PBS control) HDC−/− mice, we found only rare tryptase+ MCs in the small intestine and colon, consistent with previous data that MC frequency is decreased in the peripheral tissues of HDC−/− mice [42, 43]. In OVA treated mice, however, there was a substantial accumulation of tryptase+ intestinal MCs. Notably, the colon MC numbers were increased over 10-fold in the OVA immunized group compared to controls (Figure 1F). This was confirmed by flow cytometry (FcεRIα and c-kit staining, Supplementary Figure S1E). Collectively, these data suggest a link between HDC deficiency and the expansion of MCs and CD11b+Gr1+ cells during OVA allergy.

### MC-derived IL-17 defines HDC−/− mice OVA allergy gut immunity

**Figure 2:**
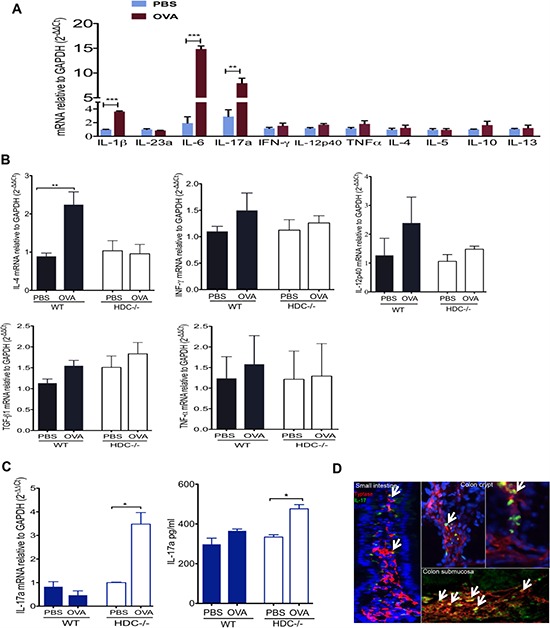
Accumulation of IL-17 producing MCs in HDC−/− mice OVA intestinal allergy **A.** Comparison of HDC−/− intestinal MC T_H_1, T_H_2, and inflammatory cytokine genes expression between OVA and PBS treated groups (*n* = 4/group). **B.** IL-4, IFN-γ, IL-12p40, TGF-β1 and TNF-α expression in WT (*n* = 3) and HDC−/− (*n* = 5) small intestine tissue, mice treated either OVA or PBS. **C.** IL-17 elevated in HDC−/− mice (*n* = 5) but not WT mice (*n* = 5) treated with OVA, bar graphs show IL-17a mRNA expression in the small intestine (Left), and IL-17a protein level in sera (Right). **D.** Representative frozen slides co-stained with tryptase and IL-17a from OVA allergy HDC−/− mice (*n* = 3) in small intestine and colon. Arrows indicate tryptase+IL-17+ MCs. Data are representative of 2–3 independent experiments.

MC cytokine secretion recruits other myeloid cells and is a major contributor to inflammation [44, 45]. Although genetic deficiency of HDC impairs granule synthesis and homeostasis of MCs [42, 46], it is unknown whether HDC/histamine status affects MC cytokine production in an allergic setting. To address this question, cytokine gene expression in MCs from HDC−/− mice treated with OVA was assessed by qRT-PCR, including T_H_1 cytokines (IFN-γ, IL-12p40, and TNFα), T_H_2 cytokines (IL-4, IL-5, IL10, and IL-13), and proinflammatory cytokines (IL-1β, IL-23a, IL-6, and IL-17a). Our results revealed that in HDC−/− mice, there was marked induction by OVA of a number of MC-derived proinflammatory cytokines, including several key cancer-promoting cytokines such as IL-1β and IL-6 and their downstream effector cytokine IL-17a (Figure 2A). Notably, these cytokines were not upregulated by OVA allergy in WT littermates (Supplementary Figure S2A). Thus, OVA treatment in the setting of histamine deficiency expanded and activated proinflammatory intestinal MCs.

To assess the overall contribution of MC-derived proinflammatory cytokines to the gut immune response to OVA allergy, and the role of histamine in modulating this response qRT-PCR analysis was performed on small intestinal tissue samples following OVA treatment. These studies revealed that, OVA allergy induced an up-regulation of T_H_2 cytokines expression in WT mice but not in HDC−/− mice (Figure 2B). Instead, we observed much greater IL-17a expression in the intestine and in the circulation of HDC−/− mice (Figure 2C). Moreover, we identified a large number of tryptase+IL17a+ MCs in the small intestine and colon of HDC−/− OVA treated mice, particularly in the colon crypts and submucosa (Figure 2D). To exclude the possibility of IL-17 production from T cells (T_H_17 or Tc17 cells) [47], we assessed IL-17a mRNA expression in sorted small intestinal CD3+CD4+ and CD3+CD8+ T cells in response to OVA treatment. IL-17a expression remained unchanged in both populations of T cells in HDC−/− mice following OVA sensitization (Supplementary Figure S2B). Although OVA treatment also increased MCs numbers in WT colon (Supplementary Figure S2C), only HDC−/− colon MCs showed increased IL-17a mRNA levels upon OVA induction (Supplementary Figure S2D). These data confirm that MCs are the key IL-17 producing cell in the intestine following OVA challenge in histamine deficient mice.

### Differentiation of IL-17-secreting MCs is regulated by histamine

**Figure 3:**
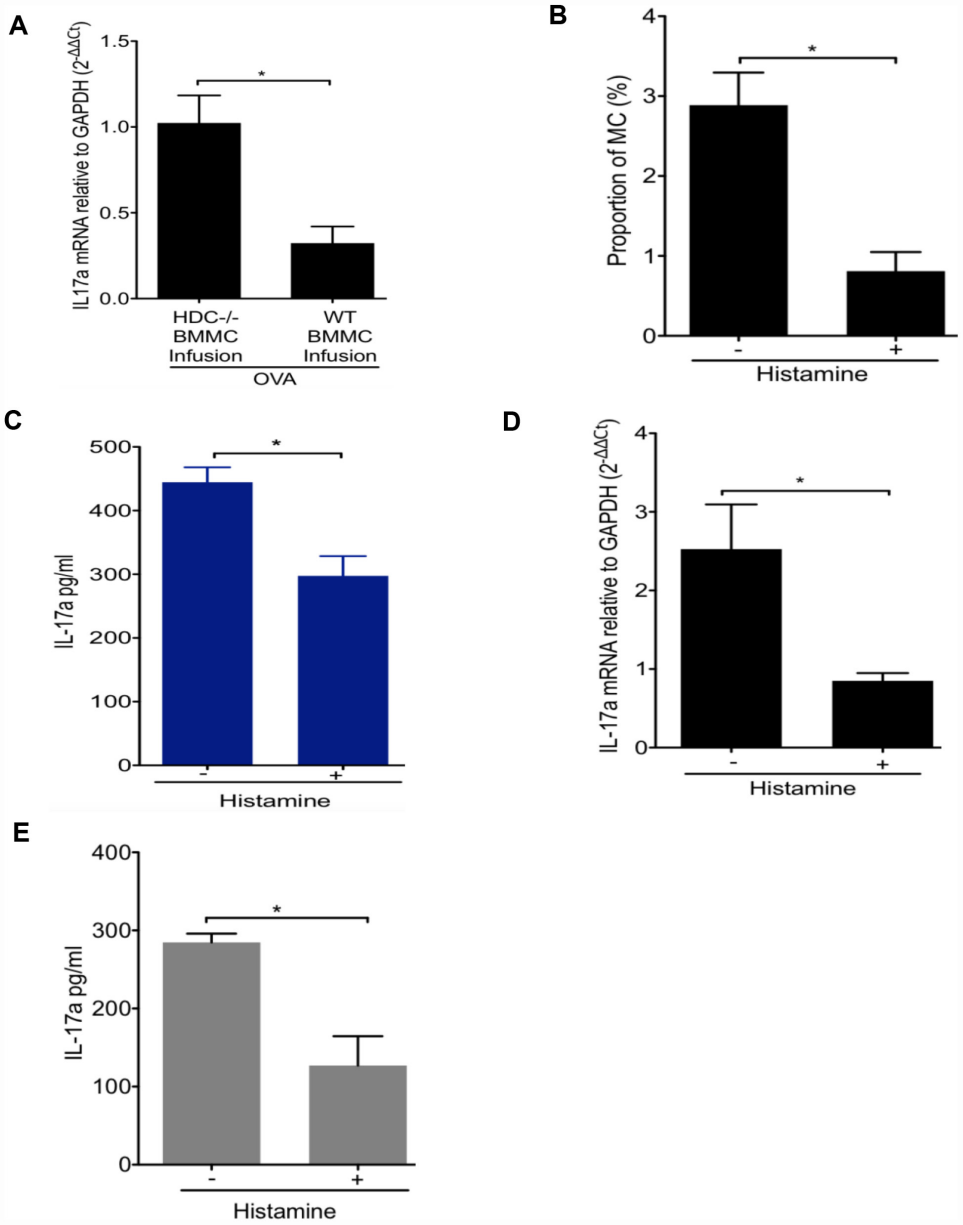
Effect of histamine on the regulation of IL-17-secreting MC **A.** HDC−/− mice (*n* = 5/group) infused with either WT or HDC−/− BMMCs followed by OVA administration. OVA allergy mice infused with WT BMMCs had decreased IL-17a expression in small intestine. **B.** Exogenous histamine treatment lower the proportion of intestinal MC (FCεRIα+c-kit+) in HDC−/− mice (*n* = 3/group) OVA allergy. **C.** Serum IL-17a levels in HDC−/− OVA immunized mice (*n* = 5/group) with or without exogenous histamine injection. **D.** IL-17a mRNA level decreased in sorted FCεRIα+c-kit+ intestinal MCs from histamine treated, OVA immunized HDC−/− mice (*n* = 5/group). **E.** *in vitro* cultured HDC−/− MC and splenocytes induced with OVA_323–339_ peptide in the presence of histamine displayed reduced IL-17a level in supernatant. Data are representative of three independent experiments.

To determine if histamine deficiency was largely responsible in the OVA allergy model for the increased production of MC IL-17, we attempted to rescue the phenotype through reconstitution with WT bone marrow-derived mast cells (BMMCs) or through exogenous administration of histamine. BMMCs from WT or HDC−/− donors were infused into non-irradiated HDC−/− mice. Daily OVA administration was begun one day after adoptive transfer, and mice were sacrificed 10 days later. Mice infused with WT MCs showed a significantly lower level of IL-17 expression in the small intestine compared to mice infused with HDC−/− MCs (Figure 3A). Exogenous histamine (5 mg/kg/d i.p.×1 mo) also significantly reduced intestinal FCεRIα+c-kit+ MCs in OVA sensitized mice (Figure 3B). Histamine treatment decreased serum IL-17 levels (Figure 3C) and small intestine MC IL-17 mRNA expression compared to the control OVA treated group (Figure 3D). We also assessed the effect of histamine on MC IL-17 production by adding 5 × 10^−6^ M histamine to HDC−/− MC co-cultured with splenocytes and OVA_323–339_ peptide. Again, we found that histamine reduced IL-17 secretion in HDC−/− MC *ex vivo* (Figure 3E). Taken together, these data suggest that histamine deficiency promotes MC expansion and production of IL-17, leading to higher serum IL-17 levels following an allergic challenge. The replacement studies establish histamine as necessary for inhibition of allergic MC accumulation and pro-inflammatory cytokine secretion.

### MC IL-17 promotes colorectal carcinogenesis in both subcutaneous and carcinogenesis models

**Figure 4:**
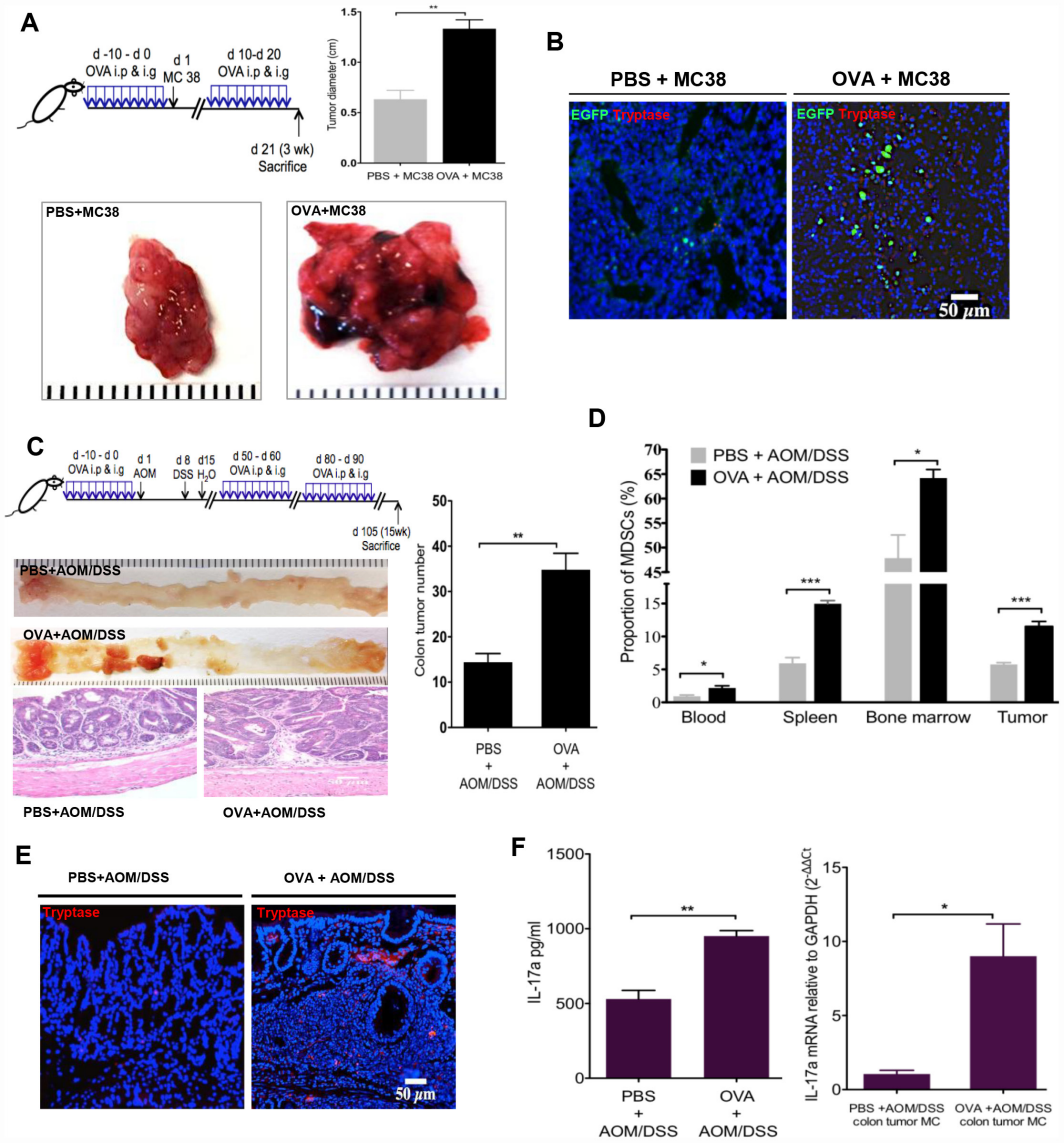
OVA intestinal allergy promote colorectal carcinogenesis in HDC−/− mice **A.** Tumor diameter from MC38 colon carcinoma tumor cells injected HDC−/−;HDC-GFP mice. Mice (*n* = 8/group) treated with OVA exhibited bigger tumor diameter than PBS controls. **B.** Increased accumulation of MCs and HDC-EGFP+ myeloid cells within MC38 carcinoma in the group of mice treated with OVA. **C.** Schematic representation of the OVA immunization plus AOM/DSS carcinogenesis protocol (Upper panel). Representative macroscopic pictures and H&E staining images from paraffin embedded colon tissue of tumor mice (*n* = 10/group, Lower panel). The number of tumors with statistics presented by bar picture (Right). **D.** Flow cytometry analysis of CD11b+Gr1+ MDSCs proportion in CD45+ cells in OVA or PBS plus AOM/DSS treated mice (*n* = 10/group) in blood, spleen, bone marrow, and colon tumor. **E.** Representative tryptase immunofluorescence staining from tumor-bearing mice colon frozen sections. **F.** OVA immunization increases serum IL-17a level and IL-17a mRNA expression in sorted tumor MCs from AOM/DSS colorectal carcinogenesis mice (*n* = 10/group). Data are representative from two independent experiments.

Recent studies have suggested that proinflammatory IL-17 has a role in promoting colorectal tumorigenesis [24, 48]. Tumor-infiltrating MCs and MDSCs are commonly found in colorectal cancer (CRC) [3, 6, 12]. As we have identified MCs as a novel cellular source of IL-17 in OVA immunized HDC−/− mice, we sought to determine whether the IL-17-secreting MCs in HDC−/− mice promote colorectal carcinogenesis and whether CD11b+Gr1+ MDSCs are involved. To investigate this, we injected MC38 (C57BL/6) colon carcinoma tumor cells subcutaneously into HDC−/−;HDC-EGFP mice. One group of mice was immunized with OVA for 10d before subcutaneous inoculation of MC38 cells, followed by repeated daily doses of OVA for days 11–21 immediately prior to harvest. The control group of mice were sham immunized with PBS. We found that mice immunized with OVA had significantly larger tumors that contained a greater proportion of both MCs and HDC-GFP+ cells (Figure 4A and 4B). These findings suggested that in the setting of histamine deficiency, colorectal cancer is promoted by allergic immune response that involve accumulation of both MCs and MDSCs.

We extended these tumorigenicity studies to a primary colorectal carcinogenesis model. We injected HDC−/− mice with 10 mg/kg of AOM, followed by 7 d of exposure to 2.5% DSS in the drinking water in order to generate colorectal tumors [28]. Before and after AOM/DSS treatment, we immunized mice with three 10d cycles of OVA or PBS. After the last cycle of OVA or PBS administration, IgE to OVA was only detectable in OVA treated group (Supplementary Figure S3A). Although both groups of mice developed colorectal tumors, OVA treated mice had significantly more colorectal tumors that exhibited higher grades of dysplasia and evidence of intramucosal carcinoma (Figure 4C). Furthermore, OVA-treated mice had a greater proportion of MDSCs in their bone marrow, spleen and circulation (Figure 4D). Notably, the number of infiltrating MCs within colorectal tumors was significantly higher in the OVA group (Figure 4E). Finally, serum IL-17a and tumor MC IL-17a mRNA levels were significantly increased after OVA plus AOM/DSS versus AOM/DSS alone (Figure 4F). Collectively, our results suggest that in histamine deficient mice, IL-17-expressing allergic MCs are capable of promoting CRC in part through recruitment of CD11b+Gr1+ cells.

### HDC−/− MCs augment CD11b+Gr1+ immunosuppressive function

**Figure 5:**
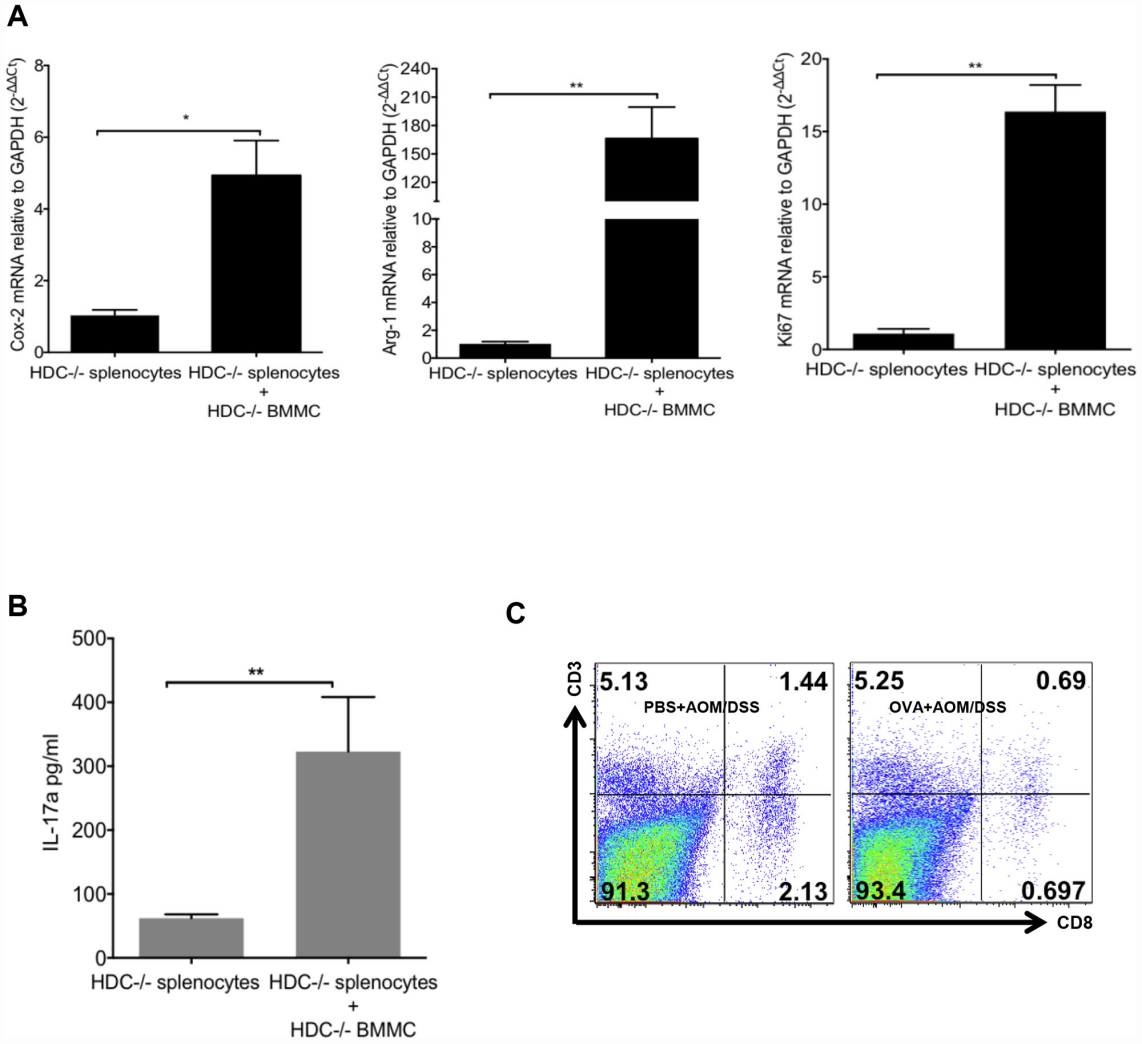
MCs support CD11b+Gr1+ MDSCs immunosuppression **A.** Co-culture of BMMCs and splenocytes from HDC−/− mice in the presence of OVA_323–339_ peptide. 96 h after culture, CD11b+Gr1+ cells were sorted, expressions of Cox-2, Arg-1 and Ki67 were determined by qRT-PCR. **B.** Supernatant IL-17 was determined by ELISA. **C.** Representative flow plots show the frequency of CD3+CD8+ cytotoxic T cell in tumor margin colon tissue (*n* = 3). Data are representative from 3 independent experiments.

Given the marked expansion of CD11b+Gr1+ cells in OVA-induced tumors above, we wondered whether these cells are the immune-suppressive MDSCs known to promote cancer. To address this, we isolated splenocytes from OVA-induced HDC−/− mice and treated the splenocytes *ex vivo* with OVA_323–339_ peptide with or without the addition of HDC−/− BMMCs. After 96 h, CD11b+Gr1+ cells were flow sorted, and the expression of cyclooxygenase-2 (Cox-2), Arg-1, and Ki67 were determined by real-time qRT-PCR. Our data confirm that in the presence of OVA, HDC−/− BMMCs were able to induce proliferation and increase expression of Cox-2 and Arg-1 in CD11b+Gr1+ cells (Figure 5A), consistent with an MDSC phenotype. Additionally, secreted IL-17 was increased in the supernatant of OVA-treated splenocytes (Figure 5B). These results confirm that IL-17 expressing MCs are able to promote the proliferation of CD11b+Gr1+ cells and enhance their immunosuppressive function through upregulation of Cox-2 and Arg-1. These data also support a potential role for OVA-induced CD11b+Gr1+ myeloid cells in mediating suppressive effects on T cells, contributing to the observed increase in colorectal cancer.

We also investigated the influence of IL-17-secreting MCs on MDSCs in the tumor microenvironment *in vivo*. We compared Cox-2, Arg-1, and Ki67 mRNA expression in tumor MDSCs in OVA immunized versus PBS-treated HDC−/− mice. Again, OVA treatment led to up-regulation of Cox-2, Arg-1 and Ki67 expression in MDSCs (Supplementary Figure S3B). Furthermore, OVA treated mice had fewer CD8+ T cells within the tumor margin compared to PBS treated controls (Figure 5C), suggesting that loss of cytotoxic T cells may be related to the promotion of carcinogenesis.

## DISCUSSION

**Figure 6:**
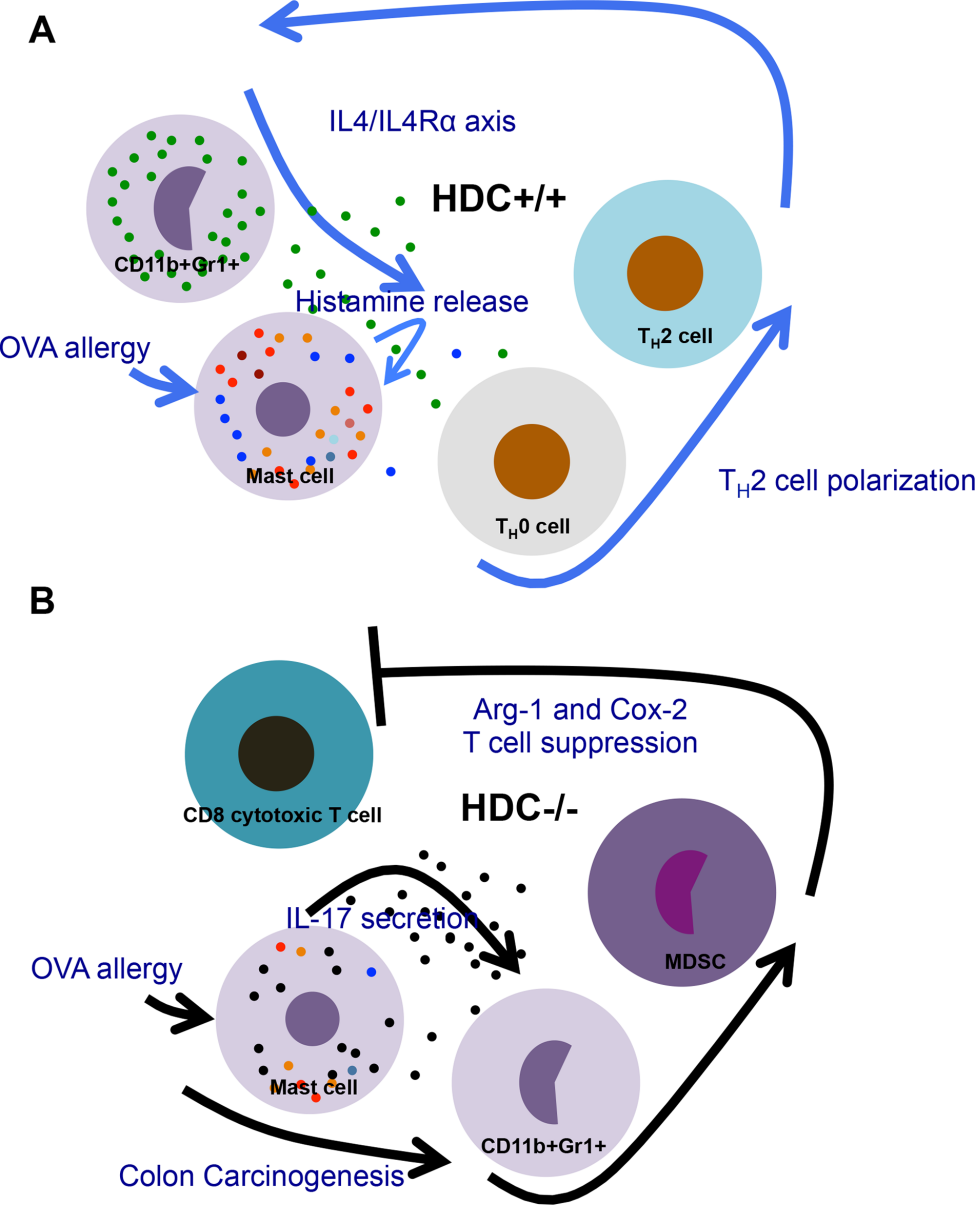
Schematic comparison of changes in HDC−/− vs WT intestine immunity during OVA allergy and following carcinogenesis **A.** In WT gut, releasing of histamine supports the normal innate allergic reactions and adoptive T helper cell activation, therefore regulates MC function through feedback manner, which maintains the homeostasis of the gut immunity. **B.** Under histamine deficiency, proinflammatory IL-17 produced by MCs augments MDSCs, in particular, upregulates Arg-1 and Cox-2 in MDSCs, thus promotes colorectal carcinogenesis.

In this study, we found in a food allergy model an expansion of IL-17 producing MCs in the intestine. In the absence of histamine-mediated negative feedback, these primed MCs promoted the expansion of MDSCs, which in turn suppressed CD8+ T cells and enhanced colorectal carcinogenesis. The production and release of histamine by MDSC and MCs, respectively, represents an important brake on MDSCs and MC in intestinal allergy (Figure 6). This may be a central mechanism in atopic patients developing colorectal cancer, and thus a potential target for prevention and therapy of intestinal malignancy.

Previous studies have demonstrated that HDC expression defines the majority of CD11b+Gr1+ MDSCs. HDC-EGFP is also expressed in the majority of IMCs in normal (non tumor-bearing) mice, and these myeloid cells represent the primary site of histamine synthesis. While MCs do not express HDC-EGFP or synthesize histamine, they take up and store histamine, and release it in response to acute allergic stimuli [28]. MCs, however, do express histamine receptors and are thought to be tightly regulated by histamine [34, 42, 43, 46]. Thus, in HDC deficient mice that have low levels of circulating histamine, we show that OVA immunization leads to an expansion of intestinal IL-17 expressing MCs. This abnormal accumulation of IL-17-secreting MCs was clearly due to histamine deficiency, since adding exogenous histamine *in vivo* reduced MC numbers and serum IL-17 levels, and also reduced IL-17 secretion in *ex vivo* cultures. In addition, these abnormalities could be rescued by adoptive transfer of MCs from HDC competent hematopoietic cells.

The inflammatory cytokine IL-17, which is significantly elevated in gastrointestinal inflammation and cancer, was originally attributed primarily to a T cell (T_H_17) response [49, 50]. However, more recently, several types of innate immune cells have been found to be additional sources of IL-17 production in the regulation of gut immunity and gastrointestinal cancer [51, 52]. In human esophageal squamous cell carcinoma (ESCC), MCs were identified as the predominant IL-17 expressing cell type by immune microscopy [23]. In the intestine of OVA immunized HDC−/− mice, the expression level of IL-17 was exclusively elevated in sorted MCs but not in either CD4 or CD8 T cells. Thus, in the setting of histamine deficiency, MC cells are polarized into IL-17 secreting cells.

IL-17 production in MCs was associated with an accumulation of MDSCs. Previous studies have suggested a causal relationship between IL-17 production and the level of circulating MDSCs [18, 50, 53]. During parasitic infection, MCs are thought to stimulate responses by immature myeloid cells, helping to mobilize CD11b+Gr1+ cells to assist with the clearance of parasites [11]. Thus, it appears that the IL-17-expressing subset of MCs expanded in the setting of histamine deficiency is particularly effective in mobilizing and recruiting cancer-promoting MDSCs to the intestinal mucosa. OVA immunization of HDC−/− mice implanted with MC38 colon cancer cells led to significantly larger tumors with increased numbers of MCs and MDSCs. In the AOM/DSS colon carcinogenesis model, OVA immunization led to increased MCs and MDSCs, and increased colorectal tumors, which correlated with a reduction in CD8+ T cells.

Tumor-infiltrating MCs have been implicated in CRC pathogenesis and are associated with a poor prognosis in human colon cancer patients [54, 55]. In mouse models of inflammation-associated colonic cancer, MCs are abundant in the tumor tissue; in contrast, MC-deficient mice are protected from colitis-associated colorectal carcinogenesis [3]. In our study, we observed an accumulation of tumor-associated MCs in response to immunization and intestinal allergy in HDC−/− mice, which supports the notion that MC acts as an important modulator in mouse enteric allergy and prolonged allergic inflammation-associated carcinogenesis.

A number of questions remain regarding the relationships between food allergy and cancer, with contrasting observations noted in epidemiologic studies [29, 30, 56–59]. On the one hand, allergic reactions strengthen host immunity that provides a strong defense against attack from invading pathogens and aberrant cells, which likely enhances cancer immune surveillance [60]. In this sense, allergic responses support protective innate immune responses and T helper cell polarization [61]. On the other hand, some studies have suggested that prolonged host allergic immunity associated with the accumulation of chronic pro-inflammatory myeloid cells might predispose to tumor development [59]. We and others have previously shown a regulatory role for histamine in the differentiation of immature innate myeloid cells. In addition, mice deficient in histamine exhibit accelerated tumor progression, and epigenetic downregulation of HDC expression in CD11b+Gr1+ cells is associated with carcinogenesis [28]. Given the pivotal role of histamine in both allergy and cancer, our results suggest that histamine-mediated MC differentiation may critically link the two disorders.

In histamine deficient gut immunity, OVA allergic MCs promote tumorigenesis through the production of the proinflammatory cytokine IL-17, expansion of MDSCs and reduced activity of tumoricidal CD8+ T cells. Restoring histamine downregulates this pathway (Figure 6). Further studies will be needed to address whether histamine or histamine analogues could be used as a novel immune modulating cancer therapy, or if histamine receptor antagonism may be detrimental in certain patients at high risk for colorectal cancer.

## MATERIALS AND METHODS

### Mice

All animals were maintained under specific pathogen-free (SPF) conditions. 6–8 week age female mice: C57BL/6 WT mice, HDC-EGFP (C57BL/6 background, HDC+/+), HDC−/− mice (C57BL/6 background) in which intron 5 and exon 6 were replaced with a neomycin cassette, and HDC−/−;HDC-EGFP (C57BL/6 background, generated by crossing C57BL/6 HDC-EGFP mice with C57BL/6 HDC−/− mice) were involved in this study [28, 42]. In OVA immunization experiment, mice were administrated orally (i.g., 30 μg) and intraperitoneally (i.p., 50 μg) without adjuvant each day for 10 consecutive days. All experiment protocols were approved by the Institutional Animal Care and Use Committee (IACUC) of Columbia University.

### Antibodies and reagents

OVA, histamine, and AOM were purchased from Sigma-Aldrich. OVA peptide (OVA_323–339_: ISQAVHAAHAEINEAGR) was obtained from AnaSpec. DSS was obtained from MP Biomedical. Anti-mast cell tryptase mouse monoclonal antibody was obtained from Abcam. Antibodies used for flow cytometry analysis and cell sorting and IL-17 immunofluorescence staining were obtained from Biolegend, including FCεRIα (MAR-1), c-kit (2B8), CD3 (17A2), CD4 (GK1.5), CD8 (53–6.7), CD11b (M1/70), Gr1 (8C5), and IL17a (TC11–18H10.1).

### Real-time quantitative RT-PCR and primers

PureLink^®^ RNA Mini Kit or RNAqueous^®^-Micro Total RNA Isolation Kit were used for tissue RNA or sorted cell RNA isolation. cDNA was synthesized by using SuperScript^®^ III First-Strand Synthesis System (Life Technologies, Grand Island, NY). Gene expression was determined using FastStart Universal SYBR Green or Probe Real-time qRT-PCR (Roche Applied Science, Indianapolis, IN). PrimeTime qRT-PCR Assays (Integrated DNA Technologies, Coralville, Iowa) were performed to measure mouse IL-17a (Mm.PT.56a.6531092) and Ki67 (Mm.PT.51.13130770) gene expression. Mouse GAPDH (Mm.PT.39a.1) was used as internal control. The sequences of primers for SYBR Green qRT-PCR are listed in Supplementary Table S1. Applied Biosystems Prism 9700 PCR machine (Applied Biosystems) was used for all real-time PCR experiments.

### Cell culture

Mouse BMMCs were differentiated *in vitro* from isolated WT or HDC−/− mice tibia and femur bone marrow cells in RPMI 1640 medium (Invitrogen) supplemented with 1 mM sodium pyruvate (Gibco), 1 mM HEPES (Invitrogen), 2 mM L-glutamine, 1% penicillin-streptomycin (Gibco), and 10% fetal calf serum (FCS, Gibco), with the presence of 1 ng/ml recombinant mouse IL-3 (Biolegend) and 10 ng/ml mouse SCF (Biolegend). The percentage of MCs was determined by flow cytometry after 4 weeks of differentiation. Purity >95% FCεRIα+c-kit+ BMMCs were subjected to further experiments. In some experiments, BMMCs were co-cultured with HDC−/− splenocytes in the presence of 10 ng/ml GM-CSF (Biolegend); 5 × 10^−6^ M histamine and/or 5 μg/ml OVA_323–339_ peptide was added respectively for additional 96 h culture.

### BMMC adoptive transfer

Differentiated BMMCs from WT or HDC−/− mice bone marrow cells were injected intravenously into syngeneic HDC−/− mice. OVA was administrated orally (30 μg) and intraperitoneally (50 μg) one day after MC transfer. 10 d after, mice were killed and exanimated form IL-17 expression.

### Flow cytometry

Intraepithelial lymphocytes (IELs) were prepared from small intestine jejunum by twice 15 min mechanical vibrating in HBSS (Gibco) medium supplemented with 5% FBS, 2 mM EDTA (Invitrogen), 0.15 mg/ml DTT (Sigma-Aldrich), and 10 mM HEPES (Invitrogen). Small intestine Lamina propria (LP), colon and colon tumor tissue were digested respectively with collagenase IV (Worthington Biochemical Corp, Lakewood, NJ), dispase II (Gibco), and DNAse I (Roche). The resulting cells were subjected to 40% and 80% Percoll gradient (GE Healthcare, Pittsburgh, PA) centrifugation to enrich the mononuclear leucocytes. Blood and spleen cells were treated with red blood cell (RBC) lysis solution (Biolegend). Cells from pooled LP and IEL, blood, spleen, and bone marrow cells were passed through a 70 μm cell strainer to prepare monolayer cell suspension for multi-color flow cytometry antibodies staining and analysis. Flow cytometry was performed on LSRII flow cytometer (BD biosciences). Data were analyzed and presented using Flowjo 9 software (Tree Star, Ashland, OR). In some experiments, FCεRIα+c-kit+, CD3+CD4+, CD3+CD8+, or CD11b+Gr1+ cells were sorted from FACSAria cell sorter (BD bioscience) for further experiments.

### ELISA

Mice were bled from the submandibular veins. Sera were separated by Microtainer tube with serum separator (BD, Franklin Lakes, NJ). The concentration of serum IL-4 or IL-17, and cell culture supernatant IL-17 were determined using mouse IL-4 or IL-17a ELISA MAX™ Deluxe kit (Biolegend) following the manufacturer's protocol. Serum OVA specific IgE was evaluated with Biolegend LEGEND MAX™ Mouse OVA Specific IgE ELISA Kit.

### Immunofluorescence staining and histological analysis

Mice small intestinal and colonic tissue samples were collected and fixed with 4% (w/v) paraformaldehyde (PFA) and cryoprotected with 30% (w/v) sucrose for making tissue frozen sections. For paraffin sections, tissue samples were fixed with 10% (w/v) formalin and dehydrated in 70% ethanol. For mast cell tryptase staining, frozen sections were first treated with Mouse on Mouse (M.O.M.) Fluorescein Kit (Vector), and then stained with primary antibody (mouse tryptase, Abcam) and secondary antibody (Goat anti-Mouse IgG, Alexa Fluor^®^ 594 conjugate, Life technologies). IL-17a was stained by a FITC-conjugated monoclonal antibody (Biolegend). Tumor paraffin sections were stained with hematoxylin and Eosin (H&E).

### Statistical analysis

Data were presented as means ± SEMs. Statistic differences between different groups were assessed by two-tailed Student's *t* test with the GraphPad Prism 5 program (GraphPad Software, San Diego, CA). *P* < 0.05 was considered to be statistically significant (*, *P* < 0.05; **, *P* < 0.01; ***, *P* < 0.001).

## SUPPLEMENTARY FIGURES AND TABLE


