# Supplementary material for: IL-17 producing mast cells promote the expansion of myeloid-derived suppressor cells in a mouse allergy model of colorectal cancer
Source: Oncotarget. 2015 Sep 26;6(32):32966–79. doi: 10.18632/oncotarget.5435 (PMC4741743; doi:10.18632/oncotarget.5435)
Supplement: Supplementary file 1 [file oncotarget-06-32966-s001.pdf]

SUPPLEMENTARY FIGURES AND TABLE

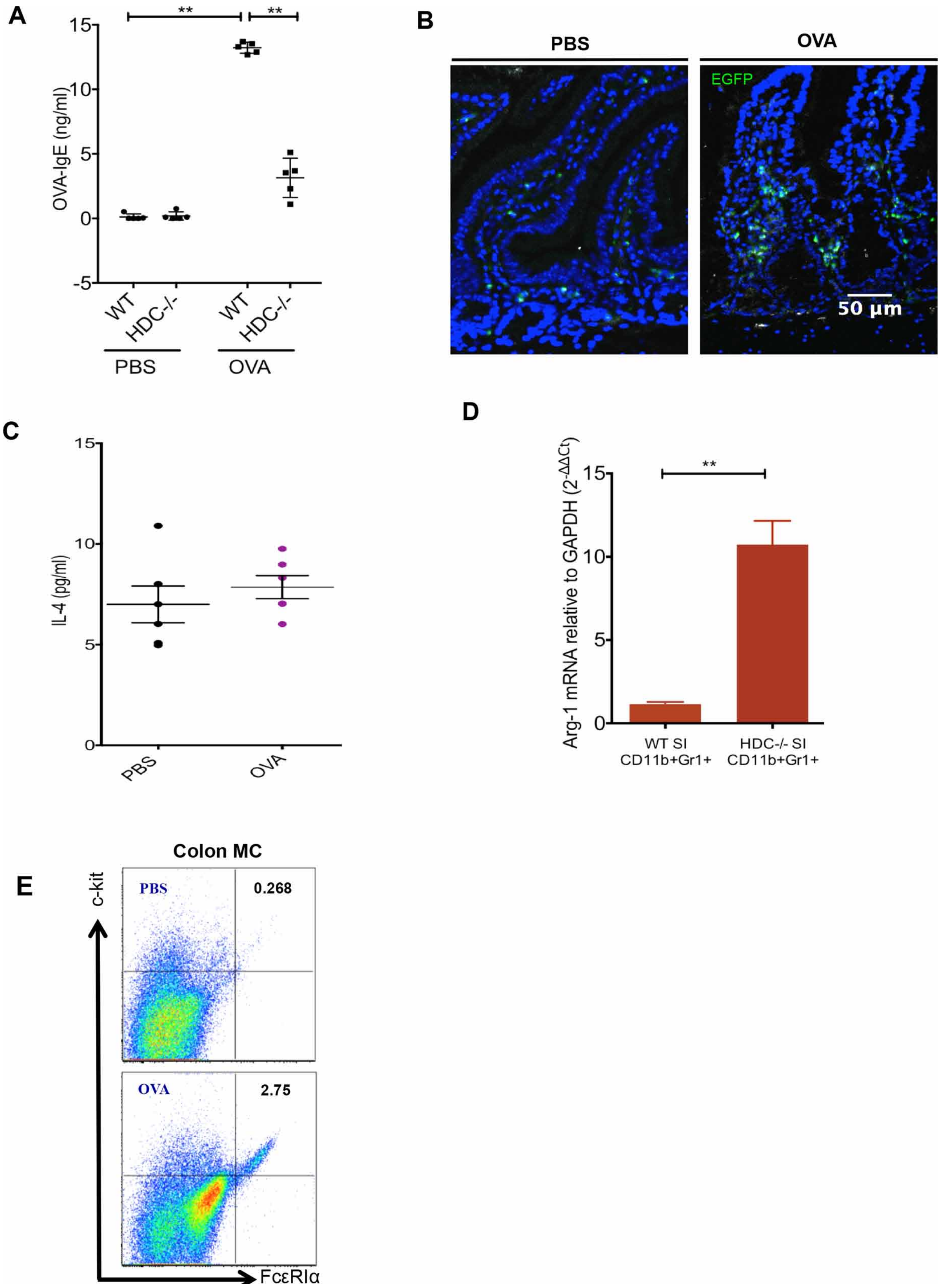

Supplementary Figure S1: Accumulation of MCs and Arg-1 hi HDC-expressed myeloid cells in HDC−/− OVA intestinal allergy. A. Serum OVA IgE levels in HDC−/− and WT mice. **B.** Accumulation of HDC+ cells in OVA treated HDC−/− mice small intestine. **C.** No elevation of serum IL-4 in HDC−/− OVA allergy mice compared to PBS controls. **D.** OVA allergy HDC−/− mice intestinal CD11b+Gr1+ cells express higher Arg-1 gene compared to WT controls. **E.** Increase of colon MCs in HDC−/− OVA allergy.

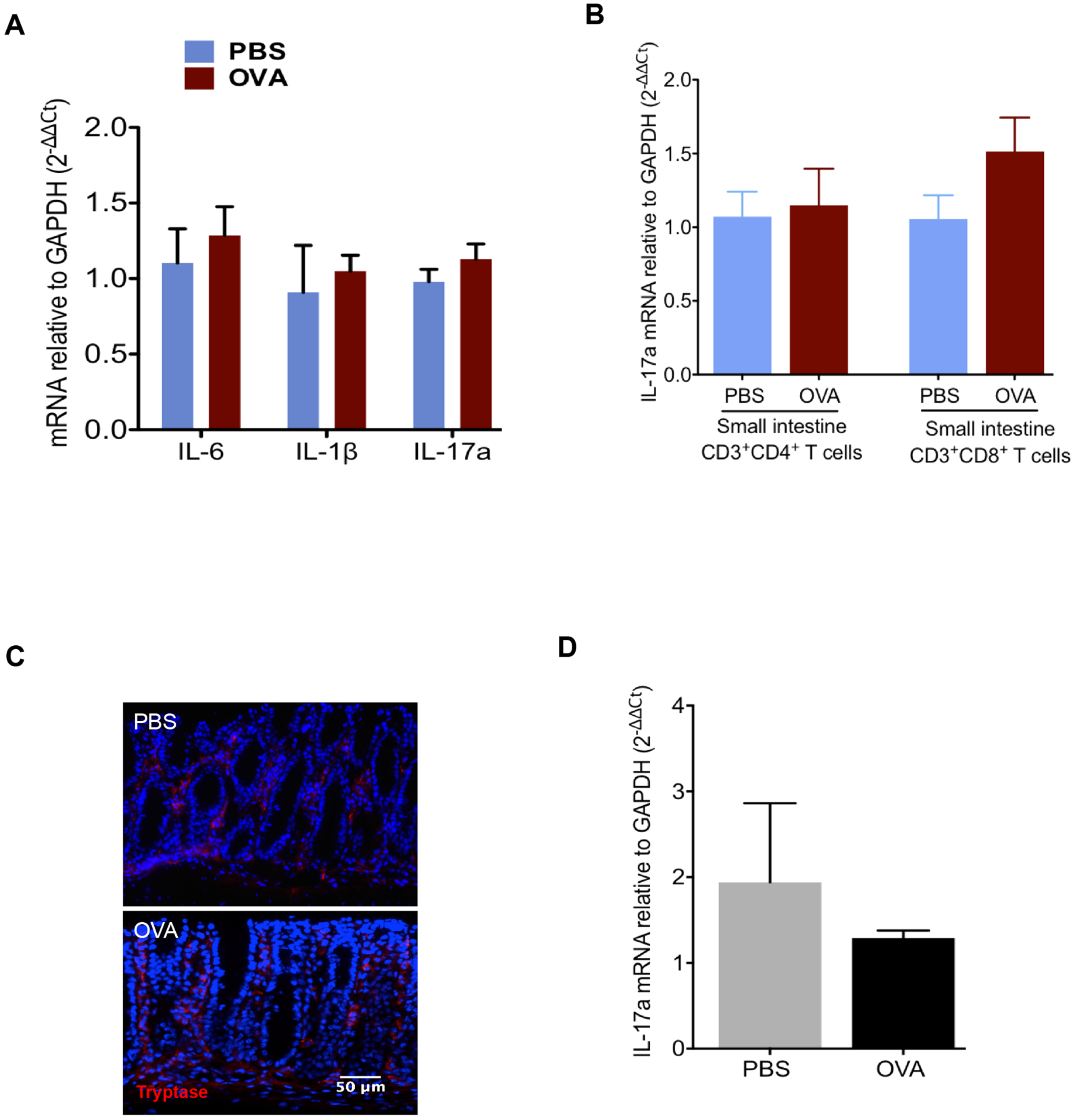

Supplementary Figure S2: MC is the IL-17 producing cell in HDC−/− OVA allergy mice intestine. A. Expression of IL-6, IL-1β, and IL-17 in WT colon MCs, compared PBS and OVA treatment. **B.** IL-17a mRNA levels in sorted CD3+CD4+ or CD3+CD8+ from PBS or OVA treated HDC−/− mice small colon. **C.** Tryptase staining on colon frozen sections from WT mice treated with PBS or OVA. **D.** IL-17a mRNA expression in WT colon MCs, compared OVA and PBS treatments.

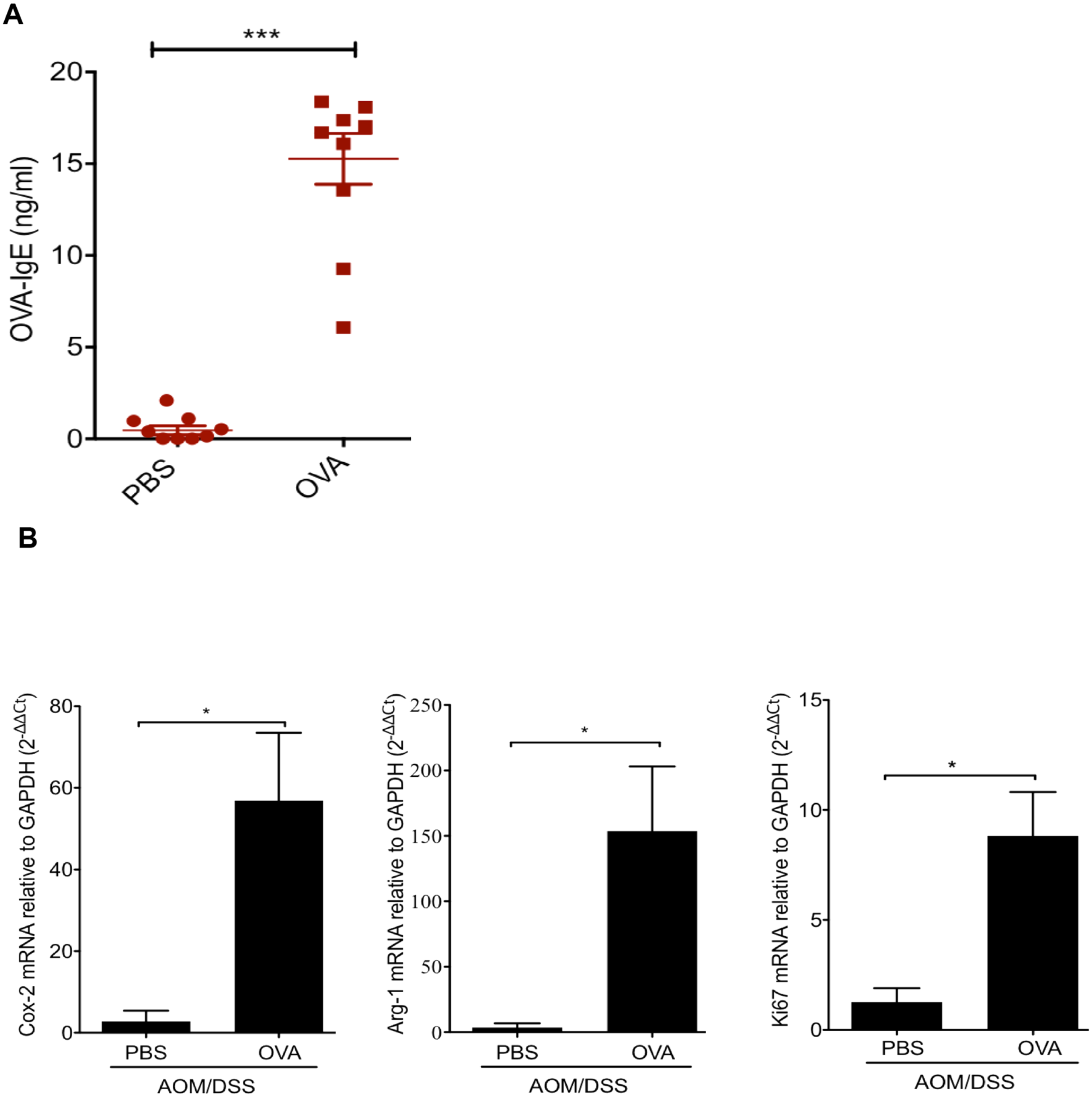

Supplementary Figure S3: MC-IL-17 augments MDSC immunosuppression in CRC. A. Presence of OVA-IgE in HDC−/− OVA allergy AOM/DSS tumor-bearing mice sera. **B.** Cox-2, Arg-1, and Ki67 expression in OVA allergy AOM/DSS tumor-bearing mice colon MDSCs.

Supplementary Table S1: Sequences of SYBR green qRT-PCR primers used in this study

Gene	Forward	Reverse
IL-1β	CGGACCCCAAAAGATGAAG	TTCTCCACAGCCACAATGAG
IL-23a	CCCGTATCCAGTGTGAAGATG	GGCTCCCCTTTGAAGATGTC
IL-6	CAAAGCCAGAGTCCTTCAGAG	GTCCTTAGCCACTCCTTCTG
INF-γ	GGCCATCAGCAACATAAGCGT	TGGGTTGTTGACCTCAAACTTGGC
IL-12p40	ACCTGTGACACGCCTGAAGAAGAT	TCTTGTGGAGCAGCAGATGTGAGT
TNF-α	TCTCATGCACCACCATCAAGGACT	ACCACTCTCCCTTTGCAGAACTCA
IL-4	AGCCATATCCACGGATGCGACAAA	AATATGCGAAGCACCTTGGAAGCC
IL-5	TGCATCAGGGTCTCAAGTATTC	GGATGCTAAGGTTGGGTATGT
IL-10	AGCCGGGAAGACAATAACTG	GGAGTCGGTTAGCAGTATGTTG
IL-13	CAGCCCTCAGCCATGAAATA	CTTGAGTGTAACAGGCCATTCT
TGF-β	GTGCGGCAGCTGTA-CATTGACTTT	TGTACTGTGTGTCCAGGCTCCAAA
Cox-2	AGTGTGCGACATACTCAAGCAGGA	TTGAAGTGGTAACCGCTCAGGTGT
Arg-1	AAGAATGGAAGAGTCAGTGTGG	GGGAGTGTTGATGTCAGTGTG
GAPDH	CTTTGTCAAGCTCATTTCCTGG	TCTTGCTCAGTGTCCTTGC
